# Ambient Ozone, PM_1_ and Female Lung Cancer Incidence in 436 Chinese Counties

**DOI:** 10.3390/ijerph181910386

**Published:** 2021-10-02

**Authors:** Huagui Guo, Jiemin Liu, Jing Wei

**Affiliations:** 1 School of Architecture and Urban-Rural Planning, Fuzhou University, Fuzhou 350108, China; huaguiguo@gmail.com; 2 Center for Global and Regional Environmental Research, Department of Chemical and Biochemical Engineering, Iowa Technology Institute, University of Iowa, Iowa City, IA 52242, USA; weijing_rs@163.com

**Keywords:** ozone, PM_1_, lung cancer, China

## Abstract

Ozone air pollution has been increasingly severe and has become another major air pollutant in Chinese cities, while PM_1_ is more harmful to human health than coarser PMs. However, nationwide studies estimating the effects of ozone and PM_1_ are quite limited in China. This study aims to assess the spatial associations between ozone (and PM_1_) and the incidence rate of female lung cancer in 436 Chinese cancer registries (counties/districts). The effects of ozone and PM_1_ were estimated, respectively, using statistical models controlling for time, location and socioeconomic covariates. Then, three sensitivity analyses including the adjustments of smoking covariates and co-pollutant (SO_2_) and the estimates of ozone, PM1 and SO_2_ effects in the same model, were conducted to test the robustness of the effects of the two air pollutants. Further still, we investigated the modifying role of urban–rural division on the effects of ozone and PM_1_. According to the results, a 10 μg/m^3^ increase in ozone and PM_1_ was associated with a 4.57% (95% CI: 4.32%, 16.16%) and 4.89% (95% CI: 4.37%, 17.56%) increase in the incidence rate of female lung cancer relative to its mean, respectively. Such ozone and PM_1_ effects were still significant in three sensitivity analyses. Regarding the modifying role of urban–rural division, the effect of PM_1_ was greater by 2.98% (95% CI: 1.01%, 4.96%) in urban than in rural areas when PM1 changed by 10 μg/m^3^. However, there was no modification effect of urban–rural division for ozone. In conclusion, there were positive associations between ozone (and PM_1_) and the incidence rate of female lung cancer in China. Urban-rural division may modify the effect of PM_1_ on the incidence rate of female lung cancer, which is seldom reported. Continuous and further prevention and control measures should be developed to alleviate the situation of the two air pollutants.

## 1. Introduction

There are great public health concerns due to the severe situation of air pollution in Chinese cities. Air pollution, already identified as the Group I carcinogenic factor to lung cancer diseases, can adversely affect human health through the increase in genetic damage [1]. According to the report of the State of Global Air 2019, fine particulate matter (i.e., PM_2.5_) was responsible for approximately three million mortalities in 2017 all over the world [2]. Despite previous efforts to tackle air pollution, especially for PM_2.5_ and PM_10_ [3,4,5], however, the associations of human health with ambient ozone and PM_1_ air pollution have not been fully understood in China.

Recently, there is increasing interest in ozone research all over China. After the implementation of clean air actions, mainly to alleviate PM_2.5_ air pollution, there has been great reductions in PM_2.5_ concentrations already, which have resulted primarily from the recent reduction in anthropogenic emissions [6,7]. Nonetheless, ozone concentrations have been increasing during the same period [8,9,10]. In parallel with the greatest public concern on PM_2.5_ air pollution, China has been a hotspot of ozone air pollution in the world. Most notably, the number of days with severe ozone situation (ozone value > 70 ppb) was higher by 93–575% in China than in most developed countries [8]. However, the nationwide study of ozone air pollution (including the nationwide estimate of ozone effects in the present study) is still in its infancy in China.

A similar increasing focus is on PM_1_ air pollution in Chinese cities. Several previous studies have suggested that PM_1_ is a major component of PM_2.5_ air pollution, accounting for the proportion of PM_2.5_ at around 80% in China [11,12]. Moreover, it has been reported that PM_1_ air pollution was more detrimental to human health than coarser PMs (e.g., PM_2.5_ and PM_10_), partly due to PM_1_’s greater surface-to-volume ratio and higher percentage of toxic chemical components [13,14,15,16]. Therefore, the understanding of PM_1_’s spatiotemporal variations and health effects is of great significance for the tailored control and prevention of air pollution in Chinese cities. Little research on PM_1_ has been performed, however, partly because of the not easily available PM_1_ data in China and the world.

As a response, few studies have estimated the effects of ozone and PM_1_ in China. In general, most of these studies suggested the positive associations of cause-specific mortalities with ozone [17,18] and PM_1_ [19,20,21]. In particular, a time-series study collecting data from 272 Chinese cities between 2013 and 2015 indicated a 0.09–0.24% higher risk of daily mortality from specific causes when 8-h maximum ozone concentrations changed by 10 μg/m^3^ [4]. Similarly, on the basis of the over-dispersed generalized additive model, another time-series analysis of 184 major Chinese cities reported that there was a positive association between ozone and pneumonia admissions [22]. With respect to PM_1_, as reported in a multi-sited study using data of daily emergency hospital visits from 26 Chinese cities, the cumulative relative risk of emergency hospital visits was 1.01 (95% CI: 1.01, 1.02) if there was a 10 μg/m^3^ increase in PM_1_ [15]. However, such nationwide studies in relation to ozone and PM_1_, are still relatively scarce in China.

Moreover, to have a more comprehensive understanding of ozone and PM_1_ effects, several interventions can be made. Firstly, of studies estimating the effect of ozone, most are single-site [17,18,23] or several-site studies [24,25]. A similar pattern of research design can be observed for PM_1_ [20,21,26]. This highlights the requirement of more nationwide studies associated with ozone and PM_1_ air pollution in China. Secondly, most ozone-related studies focus on cause-specific morality [25,27], while little attention has been placed on the morbidity or incidence. Thirdly, much ozone-associated research targets the cause of respiratory and cardiovascular diseases [4,28,29], whereas limited research focuses on other causes [21,30]. Fourthly, few studies examine socioeconomic modification effects on the association of human health with air pollution. The understanding of the socioeconomic modifying role on the health effect of air pollution is highly essential for policy making to develop strategies that are tailored for specific locations and people to reduce health disparities. Despite such significance, however, it is still unclear whether socioeconomic factors modify the effects of air pollution, especially for ozone and PM_1_.

To fill the aforementioned gaps, this study aims to estimate the effect of ambient ozone and PM_1_ on the incidence rate of female lung cancer using data collected from 436 cancer registries (counties/districts) of China during 2014–2016. We also examined whether the effects of the two air pollutants found are robust to the controls of smoking factors and additional air pollutants and are sensitive when estimating ozone, PM_1_ and SO_2_ effects in the same model. Moreover, we further investigated whether the effects of ozone and PM_1_ differ between urban and rural areas in China.

## 2. Data and Methods

### 2.1. Research Area

**Figure 1:**
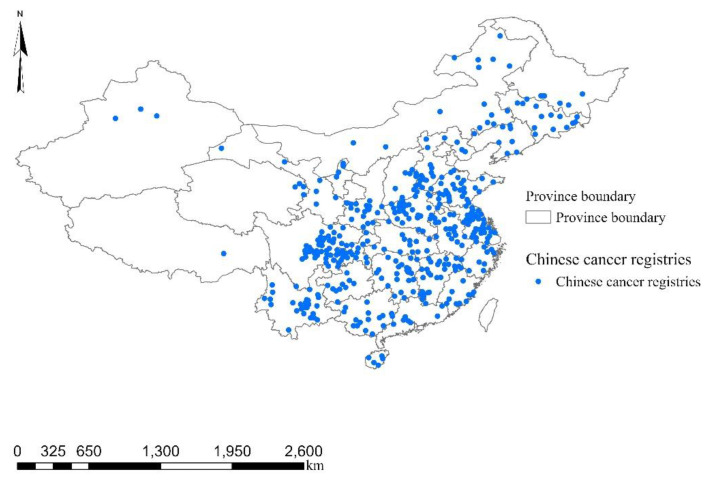
Spatial distributions of 436 Chinese cancer registries during 2014–2016.

As shown in Figure 1, our research area covered 436 Chinese cancer registries dispersed over 31 of 34 Chinese province-level administrative regions. Of these registries, they are 326 rural registries (counties), and 110 urban registries (districts). The 436 registries (counties/districts), home to around 272.12 million residents in 2016, were selected on the basis of the availability of data on air pollution (2014–2020), lung cancer (2006–2016) and socioeconomic covariates (2006–2016). The annual mean ozone and PM_1_ concentrations of 436 registries in 2016 were 86.76 μg/m^3^ and 30.32 μg/m^3^, respectively.

### 2.2. Data Collection

#### 2.2.1. Ambient Ozone and PM_1_

**Figure 2:**
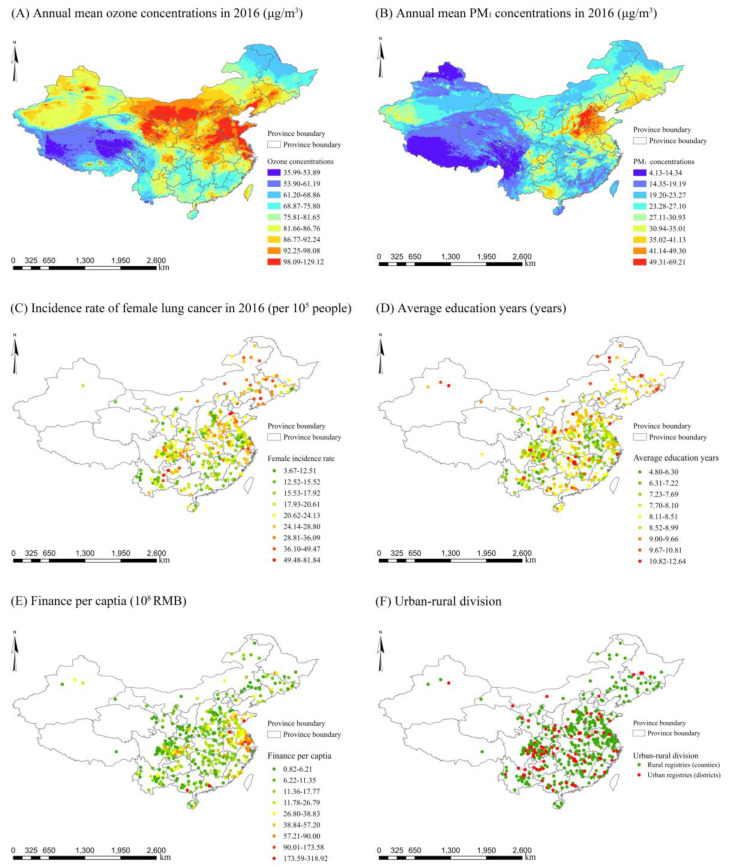
Spatial distributions of annual mean ozone and PM_1_ concentrations, the incidence rate of female lung cancer and some socioeconomic covariates.

Data on annual surface ozone concentrations at 10 km × 10 km grids between 2014 and 2016 were acquired from the ChinaHighO_3_ dataset (http://doi.org/10.5281/zenodo.4400043 (accessed on 30 December 2020)). This dataset is one of the full-coverage and high-quality datasets of surface air pollution in China. Technologically, the production of surface ozone data was similar to that of the ChinaHighPM_2.5_ dataset [31,32]. In brief, the surface ozone concentrations were estimated using the proposed Space-Time Extra-Trees (STET) model (i.e., one of the most advanced machine learning methods in the field of remote sensing estimation of air pollution concentrations). Data as model inputs mainly included the MAIAC (Multi-Angle Implementation of Atmospheric Correction) AOD (aerosol optical depth) product, meteorological factors, surface conditions (e.g., Normalized Difference Vegetation Index (NDVI) and land use cover), pollutant emissions and population distributions, as well as the terms capturing the spatial and temporal autocorrelations of surface ozone concentrations. As reported (http://doi.org/10.5281/zenodo.4400043 (accessed on 30 December 2020)), there is high consistency between the estimated ozone data and ground-based ozone measurements (R^2^ = 0.87, root-mean-square error (RMSE) = 17.10 μg/m^3^). Figure 2A presents the spatial distributions of ozone concentrations across China in 2016.

Data on annual PM_1_ concentrations at 1 km^2^ spatial resolution between 2014 and 2016 were obtained from our previous study. More details on PM_1_ estimates can be found in Wei et al. [33]. Briefly, a machine learning approach, i.e., the space-time extremely randomized trees model (STET), was developed to estimate daily PM_1_ concentrations in terms of the geospatial data of MAIAC AOD, MEIC pollution emissions, meteorological factors, land use, road and population, as well as the spatiotemporal information which captures the spatial and temporal autocorrelations of PM_1_ concentrations. According to the results from model validation, there is high agreement between the estimated daily PM_1_ concentrations and ground-level PM_1_ measures, with R^2^ and RMSE equal to 0.77 and 14.6 μg/m^3^, respectively [33]. Such high agreement could also be seen when PM_1_ was estimated at the seasonal and annual scales, with R^2^ equal to 0.97 and RMSE lower than 4.1 μg/m^3^. To date, this dataset has been increasingly used to estimate health burden [34] as well as the effects of PM_1_ on human health in Chinese cities [21,35,36]. The spatial distributions of PM_1_ concentrations in 2016 all over China are shown in Figure 2B.

#### 2.2.2. Incidence Rate of Female Lung Cancer

Annual age-standardized incidence rates of trachea, bronchus and lung cancer for females (i.e., the incidence rate of female lung cancer hereafter) between 2014 and 2016 were acquired from the 2017–2019 China Cancer Registry Annual Reports [37,38,39]. This health outcome, defined as the incidence number of lung cancer for females per 100,000 people per year in a registry (county/district), was age-standardized in terms of Segi’s world population. The annual reports used in the present study were publicly released by the Chinese Cancer Registry at the National Cancer Centre of China with the aim of providing timely, representative and comprehensive information on cancer diseases from specific causes all over China (including the incidence rate of female lung cancer in the present study). For example, data on cause-specific cancer diseases for each of the 682 Chinese cancer registries were reported in the 2019 China Cancer Registry Annual Report [39]. These registries were located in 31 of the 34 province-level administrative regions in China. Figure 2C exhibits the spatial distributions of the incidence rate of female lung cancer in 2016.

#### 2.2.3. Socioeconomic Characteristics and Smoking Covariates

Six socioeconomic covariates were chosen according to their reported associations with lung cancer diseases in prior studies [24,40,41]. These covariates included finance per capita, population, average education years, proportions of construction and manufacturing workers, and urban–rural division. Data on the first two variables were derived from the 2015–2017 China Statistical Yearbook (County-Level), while other data were obtained from the tabulation of the 2010 population census of the People’s Republic of China. Figure 2D–F show the spatial distributions of educational attainment, financial level and urban–rural attributes for 436 Chinese cancer registries (counties/districts), respectively.

Data on smoking covariates were acquired from the 2015 China Health and Retirement Longitudinal Study (CHARLS) wave3. This publicly accessible dataset was released by the National School of Development, Peking University (http://charls.pku.edu.cn/en/page/data/2015-charls-wave4 (accessed on 31 May 2017)). The CHARLS survey aims to provide the timely information on socioeconomic and health conditions of Chinese residents who are 45 years and older across China, which is therefore representative and comprehensive at the national scale [42]. There are 10,257 households and 17,708 individuals recruited by the CHARLS survey with a spatial coverage of 28 Chinese province-level administrative regions [42]. To date, this dataset has been used to identify the influential factors of mental and physical health [43,44,45].

### 2.3. Statistical Analysis

The effects of ozone and PM_1_ on the incidence rate of female lung cancer were estimated using two linear regression models. In Model 1, we solely included air pollutant (i.e., ozone and PM_1_), time and location factors. Notably, the effect of ozone (and PM_1_) is expressed as the change in the incidence rate of female lung cancer relative to its mean when ozone (and PM_1_) changed by 10 μg/m^3^. In Model 2, we further adjusted for socioeconomic factors, including finance per capita, average education years, proportions of construction and manufacturing workers, population and urban–rural division (as a dummy variable). These socioeconomic covariates were selected primarily as a result of their connections with lung cancer diseases reported in previous studies [24,40,41].

Then, we performed three sensitivity analyses to test the sensitiveness of ozone and PM_1_ effects. Firstly, we tested whether the effects of the two air pollutants are still significant after the adjustment of smoking covariates. Smoking prevalence (shortened to smoking_p) in combination with smoking strength (shortened to smoking_s, namely, the number of cigarettes smoked per day), was selected according to the reported connections of lung cancer diseases with the two smoking covariates in prior studies [46]. Notably, the CHARLS-derived smoking dataset is available at city level and does not cover all counties/districts of the present study. As a response, the same smoking characteristics were attributed to counties/districts located in the same city, which left around 48% of the whole sample. Based on the dataset of around 48% of the whole sample, we examined the effects of ozone and PM_1_ in the two situations, i.e., with and without the controls of smoking factors.

Secondly, we tested whether the effects of ozone and PM_1_ found in the present study are sensitive to the further control of additional air pollutant (i.e., SO_2_). The additional air pollutant is measured as the annual mean SO_2_ concentrations aggregated in each county/district. The SO_2_ data at 0.5° × 0.625° spatial resolution were acquired from the time-series dataset of M2TMNXAER (V5.12.4), which is freely published by the Global Modelling and Assimilation Office of NASA in USA. Details about the production of the SO_2_ dataset can be found in Randles et al. [47] and Buchard et al. [48]. Currently, the SO_2_ dataset has been increasingly used in air pollution studies [49,50]. Thirdly, we examined whether the effects of ozone and PM_1_ are still significant when estimating the effects of these two targeted air pollutants and additional air pollutant (i.e., SO_2_) in the same model. One of the aims is to determine the relative importance of the three air pollutants in increasing the incidence rate of female lung cancer.

Finally, we investigated the modification effect of urban–rural division on the association between ozone (and PM_1_) and the incidence rate of female lung cancer. Firstly, the whole dataset was stratified according to urban–rural division. Following the method of urban and rural division adopted in the cancer registry annual report as well as many previous studies conducted in China [39,51,52], the present study used counties and districts to represent rural and urban areas, respectively. We conducted the comparison of the effects of ozone (and PM_1_) between urban and rural groups in terms of Model 2. Then, the stratified dataset was combined, and the interaction of ozone (and PM_1_) with urban–rural dummy variable was added to Model 2; this was used to investigate whether the effect of air pollution significantly varies between urban and rural areas. Notably, the urban–rural dummy variable was not included in the model because this variable had a high collinearity with its interaction (i.e., the interaction between urban–rural dummy variable and ozone (and PM_1_)).

## 3. Results

### 3.1. Descriptive Analysis

Table 1 presents the descriptive statistics of health outcome, air pollutants and socioeconomic factors for 436 Chinese cancer registries (i.e., counties/districts). The mean incidence rate of female lung cancer for 436 Chinese counties/districts was 22.42 per 10^5^ people. As shown in Table 1, a great variation in the incidence rate of female lung cancer among 436 counties/districts was also observed, with the standard deviation of 8.85. With regard to the two air pollutants, the mean values of ozone and PM_1_ were 84.32 μg/m^3^ and 34.67 μg/m^3^, respectively. The values of the two air pollutants also varied considerably all over the counties/districts (Table 1). Such large variations were also observed for socioeconomic covariates (Table 1).

### 3.2. Effects of Ambient Ozone and PM_1_

The results of the spatial association between ozone and the incidence rate of female lung cancer are shown in Table 2. In general, there was a positive effect of ozone. As shown in Table 2, if there was a 10 μg/m^3^ increase in ozone, then the change in the incidence rate of female lung cancer relative to its mean was 4.91% (95% CI: 5.46%, 16.54%) in Model 1. With the control of socioeconomic characteristics in Model 2, there was a slight decrease in the effect of ozone. Specifically, a 10 μg/m^3^ increase in ozone was positively associated with a 4.57% (95% CI: 4.32%, 16.16%) increase in the incidence rate of female lung cancer relative to its mean (Table 2).

Table 3 exhibits the results of the effect of PM_1_ on the incidence rate of female lung cancer. Generally, there were positive associations of the female lung cancer incidence rate with PM_1_. In Model 1 without socioeconomic controls, the change in the incidence rate of female lung cancer relative to its mean was 4.60% (95% CI: 3.91%, 16.70%), when PM_1_ changed by 10 μg/m^3^. A similar pattern of results was observed in Model 2. Specifically, as shown in Table 3, if there was a 10 μg/m^3^ increase in PM_1_, the change in the incidence rate of female lung cancer relative to its mean was 4.89% (95% CI: 4.37%, 17.56%).

### 3.3. Sensitivity Analysis

#### 3.3.1. Control of Smoking Factors

**Figure 3:**
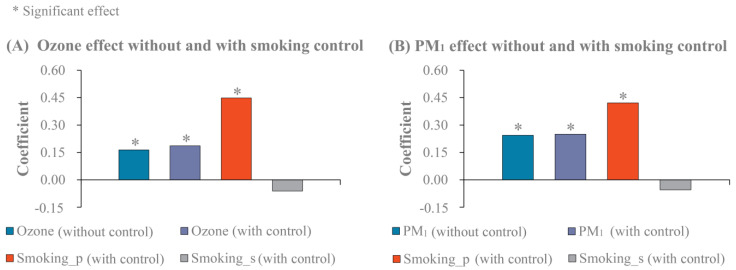
Sensitivity analyses of ozone and PM_1_ effects to the adjustment of smoking covariates. Smoking_p and smoking_s denote smoking prevalence and smoking strength (i.e., the number of cigarettes smoked per day), respectively.

Sensitivity analyses of air pollution effects to the control of smoking characteristics are shown in Figure 3. In general, ozone and PM_1_ effects were not sensitive to the smoking control. As shown in Figure 3A, there was a positive association between ozone and the incidence rate of female lung cancer without the adjustment of smoking factors. When further controlling for smoking covariates, the effect of ozone was still significant (Figure 3A); smoking prevalence was also positively correlated with the incidence rate of female lung cancer (Figure 3A). A similar pattern of results was observed for PM_1_ (Figure 3B). In particular, both PM_1_ and smoking prevalence exerted their significant effects on the incidence rate of female lung cancer after the adjustment of smoking factors (Figure 3B).

#### 3.3.2. Adjustment of Additional Air Pollutant (i.e., SO_2_)

Table 4 presents the results of the sensitivity analysis when SO_2_ (i.e., the additional air pollutant) is controlled. In general, the effects of ozone and PM_1_ were robust to the adjustment of additional air pollutant (i.e., SO_2_). As shown in Table 4, a 10 μg/m^3^ increase in ozone was positively associated with a 3.95% (95% CI: 2.89%, 14.84%) increase in the incidence rate of female lung cancer relative to its mean; notably, the effect of SO_2_ on the incidence rate of female lung cancer was also significant (β = 3.34%, 95% CI: 2.35%, 12.63%) (Table 4). A similar pattern of results was seen for PM_1_ (Table 4). In particular, as presented in Table 4, there were positive associations of the incidence rate of female lung cancer with PM_1_ and SO_2_.

#### 3.3.3. Ozone, PM_1_ and Additional Air Pollutant (SO_2_) Effects in the Same Model

The results of the sensitivity analysis estimating ozone, PM_1_ and additional air pollutant (SO_2_) effects in the same model are presented in Table 5. In general, the effects of the three air pollutants were still significant. Without the adjustment of socioeconomic covariates, each air pollutant, including ozone, PM_1_ and SO_2,_ was positively associated with the incidence rate of female lung cancer; the greatest effect size was for ozone, followed by PM_1_ and SO_2_. A similar pattern of results was observed when socioeconomic factors were adjusted. In particular, as shown in Table 5, the association with the incidence rate of female lung cancer was strongest for ozone (β = 3.94%, 95% CI: 1.28%, 6.60%), followed by PM_1_ (β = 3.45%, 95% CI: 0.28%, 6.61%) and SO_2_ (β = 2.32%, 95% CI: −0.15%, 4.79%).

### 3.4. Modification Effect of Urban–Rural Division

Table 6 and Table 7 exhibit the results of urban–rural modification effect on the association between ozone (and PM_1_) and the incidence rate of female lung cancer. Generally, urban–rural division (rural group as the reference group) positively modified the effect of PM_1_. In the stratified dataset, as shown in Table 6, the effect of PM_1_ was larger in urban than in rural areas with the coefficients of 0.11 and 0.08, respectively; in the combined dataset, as shown in Table 7, the change in the incidence rate of female lung cancer relative to its mean was higher by 4.14% (95% CI: 2.68%, 15.88%) in urban than in rural areas if there was a 10 μg/m^3^ increase in PM_1_. With regard to ozone, however, no modifying role of urban–rural division was observed. Specifically, despite the significant effect of the interaction between ozone and urban–rural dummy variable, ozone was not positively associated with the incidence rate of female lung cancer in the urban group (Table 6).

## 4. Discussion

Despite the implementation of multiple air-clearing actions, ozone concentrations have been increasing, and the magnitude and frequency of severe ozone air pollution were both higher in China than in most developed countries. Ozone has now become another air pollutant threating human health in Chinese cities on which to focus. PM_1_ is likely to be more detrimental to the human body than coarser PMs (e.g., PM_2.5_ and PM_10_). Meanwhile, lung cancer has already become the second-most common type of cancer incidences for females in China, with the incidence rate of female lung cancer at 42.28 per 100,000 people in 2016 [39]. Despite such significance, however, few nationwide studies have investigated the effects of the two prominent air pollutants on the incidence rate of female lung cancer in China. As one of the earliest attempts in China, this nationwide study estimated the effects of ozone and PM_1_ in 436 cancer registries (counties/districts) of China between 2014 and 2016.

We found a positive effect of ozone on the incidence rate of lung cancer. This is in line with the findings from previous Chinese and Western studies. In particular, a nationwide study acquiring data from 75 Chinese counties/districts between 1990 and 2009 indicated that ozone was positively associated with the incidence rate of lung cancer in the spatial age-period-cohort model [51]. Similarly, using data of 22.2 million US Medicare beneficiaries from 2000 to 2008, another nationwide study indicated the adverse effect of ozone exposure on lung cancer-associated mortality in the United States [53]. The positive associations of lung cancer diseases with ozone were also reported in other studies [54,55].

We found a greater effect of PM_1_ on the incidence rate of female lung cancer in urban than in rural areas. This finding is consistent with those of previous studies in relation to the modifying role of socioeconomic factors on the effects of PMs. As indicated in our previous study, the increase in the incidence rate of male lung cancer relative to its mean was larger by 2.47% (95% CI: 0.38%, 4.55%) in urban areas than in rural areas when PM_1_ changed by 10 μg/m^3^ [50]. Such findings regarding urban–rural division’s modifying role on PM effects were further enhanced by many other studies [41,51,56]. For example, when there was a 10 μg/m^3^ increase in PM_2.5_ in a nationwide study of China, the relative risk of lung cancer incidence for urban areas was 1.06 (95% CI: 1.04, 1.08), which is higher than 1.04 (95% CI: 1.00, 1.08) for the rural group [51]. Theoretically, three differences (i.e., material resources, biological factors and psychological stress) may be responsible for the varying effects of air pollution among different socioeconomic groups.

In the present study, as discussed in our previous studies [50,57], the difference in smoking behaviours between urban and rural areas may explain the differential effects between the two groups. Briefly, there were higher cigarette-associated indicators, including smoking strength (i.e., the number of cigarettes smoked per day) and the prevalence of cigarette smoking among all smokers, in urban than in rural areas of China [58]; at the same time, the age at which people began regularly smoking was also younger in urban than in rural groups of China [58]. The more severe smoking-associated situation in urban areas may explain the higher smoking-related hazard risks (e.g., lung cancer incidence and mortality) in urban than in rural areas [58]. This may cause people living in urban areas to be more vulnerable to exposure to air pollution (including PM_1_), and thus more greatly affected by PM_1_, since previous studies have reported the differential effects of air pollution on the physical health of human beings among people having different smoking behaviors [59,60]. The finding in the present study supports the argument that socioeconomic factors can modify the association between air pollution and human health (including physical and mental health).

There are several strengths in the present study. Firstly, this is one of the few large-scale studies in China with data collected from 436 Chinese counties/districts. This nationwide study provides representative and comprehensive evidence of the effects of ozone and PM_1_ from a developing setting (i.e., China) where the magnitude and frequency of severe ozone pollution are all larger than those of most developed countries. Secondly, this study focuses on ozone air pollution, which has become another health risk in Chinese cities on which to focus. We also pay attention to PM_1_ which accounts for a large proportion of the dominant PM_2.5_ air pollution in China. Thirdly, collecting data from 436 registries (counties/districts) in the present study enables us to further investigate modification effects of socioeconomic factors (i.e., urban–rural division) in the setting of China where urban–rural division is highly prominent.

By contrast, several limitations and prospects should be well noted and discussed. Firstly, exposure to air pollution was operationalized as the registry-aggregated concentrations of ozone (and PM_1_), and thus did not consider individual mobility. Hence, as in most prior ecological studies related to air pollution [4,24,61], there are errors in exposure measurements of the two air pollutants in the present study. Secondly, it is not feasible for us to examine the lag effects of ozone and PM_1_ on the incidence rate of female lung cancer in this work. Some studies have pointed to the lag effects of air pollution, including the single- and moving-average lags [50,57,62]. However, our data with respect to ozone and PM_1_ are available from 2014 and after (female lung cancer data are available from 2006 to 2016), so it is not feasible to investigate the potential long-latency of lung cancer development associated with exposure to ozone and PM_1_. If data on the two air pollutants prior to 2014 are available, this limitation should be well addressed and handled.

Thirdly, similar to those of our previous studies [50,57], the lack of smoking data at county/district level may make our findings concerning air pollution effects sensitive to the control of smoking covariates. As a response, we derived city-level smoking data from the CHARLS survey (which left around 50% of the whole registries for the sensitivity analysis) to test whether the effects of ozone and PM1 are still significant with the control of smoking factors. Such operationalization has two main limitations. On the one hand, our findings acquired from the whole registries may still not be robust to the control of smoking factors, although the sensitivity analysis using the dataset of around 50% of the whole registries has shown the robustness of air pollution effects to the control of smoking factors. One the other hand, CHARLS-derived smoking data are available at city level, so counties/districts located in the same city were attributed with the same smoking characteristics in the present study. This operationalization suffers from problems such as ecological fallacy. If data on county-level smoking covariates are available, such limitations should be well considered and thus addressed.

Fourthly, the uneven distribution of cancer registries inherited from the China Cancer Registry Annual Report may bias the estimate of air pollution effect on the incidence rate of female lung cancer in the present study. Although the cancer registries included in the annual report are dispersed over 31 of 34 Chinese province-level regions, they are not evenly distributed. That is, most registries (counties/districts) are concentrated in Southeast China, while registries are quite scarce in the west of China. Such uneven distribution inherited from original health data is likely to bias the effect estimate in the present study. Fifthly, despite some efforts [15,19,63], it is still not sufficient to conclude that finer particulate matters have greater effects on human health (physical and psychological health), especially in developing countries where data of PMs (especially for PM_1_) are usually scarce or quite limited. This highlights the great need to examine the effects of multiple PMs (e.g., PM_1_, PM_2.5_ and PM_10_) at a nationwide scale in the future.

## 5. Conclusions

Ozone and PM_1_ were positively associated with the incidence rate of female lung cancer in China. Moreover, urban–rural division may modify the association of the incidence rate of female lung cancer with PM_1_, with a higher effect of PM_1_ observed in urban areas. There was no modifying role played by urban–rural division for ozone. The implications of our findings are two-fold. On the one hand, this study suggests that the continuous clear-air actions in China, especially the strict prevention and control strategies of air pollution, should be well designed to consider not only the dominant PM_2.5_ air pollution in China, but also PM_1_ and ozone which has become another focus of health risks in Chinese cities. On the other hand, area-specific measures, such as reducing the disparities in access to healthcare resources between urban and rural areas, should be well considered and developed to reduce urban–rural disparities in the health effects of air pollution (especially for PMs) in China.

## Figures and Tables

**Table 1:** Descriptive analysis of health outcome, air pollutants and socioeconomic covariates for 436 counties (districts).

Variables	Mean	SD	Min	Max
Incidence rate of female lung cancer (per 10^5^ people)	22.42	8.85	0.00	81.84
Ozone (μg/m^3^)	84.32	10.64	46.68	108.93
PM_1_ (μg/m^3^)	34.67	11.14	8.56	71.67
Finance per capita (10^8^ RMB)	23.34	31.12	0.81	318.92
Average years of education (years)	8.40	1.20	4.80	12.64
Construction workers% (10^−2^)	31.99%	21.78%	4.25%	314.47%
Manufacturing workers% (10^−2^)	79.84%	78.17%	2.48%	421.04%
Population (10^4^ people)	64.33	35.29	4.00	186.23

**Table 2:** Ozone and the incidence rate of female lung cancer: associations in the two models.

Variables	Model 1		Model 2	
	β	95% CI	β	95% CI
Ozone	4.91% ***	(5.46%, 16.54%)	4.57% ***	(4.32%, 16.16%)
Latitude	−0.85 **	(−1.62, −0.08)	−0.87 **	(−1.66, −0.07)
Latitude^2^	0.02 ***	(0.01, 0.03)	0.02 ***	(0.01, 0.03)
Year 2015	−1.14 *	(−2.47, 0.19)	−1.30 **	(−2.62, 0.03)
Year 2016	−0.72	(−2.01, 0.58)	−0.86	(−2.16, 0.43)
Finance			0.03 ***	(0.01, 0.06)
Education			−0.84 ***	(−1.42, −0.26)
Construction			−0.04 ***	(−0.06, −0.01)
Manufacturing			0.00	(−0.01, 0.01)
Population			0.01	(−0.01, 0.03)
Urban–rural division			1.94 **	(0.34, 3.55)

* for *p* < 0.1, ** for *p* < 0.05 and *** for *p* < 0.01. If ozone changes by 10 μg/m^3^, then the change in the incidence rate of female lung cancer relative to its mean = (10× ozone coefficient)/mean incidence rate.

**Table 3:** PM_1_ and the incidence rate of female lung cancer: associations in the two models.

Variables.	Model 1		Model 2	
	β	95% CI	β	95% CI
PM_1_	4.60% ***	(3.91%, 16.70%)	4.89% ***	(4.37%, 17.56%)
Latitude	−0.71 **	(−1.48, 0.05)	−1.01 **	(−1.83, −0.18)
Latitude^2^	0.02 ***	(0.01, 0.03)	0.02 ***	(0.01, 0.03)
Year 2015	−0.26	(−1.75, 1.23)	−0.32	(−1.81, 1.17)
Year 2016	1.00	(−0.51, 2.52)	0.91	(−0.60, 2.43)
Finance			0.03 **	(0.01, 0.06)
Education			−1.16 ***	(−1.75, −0.57)
Construction			0.03 **	(−0.06, 0.00)
Manufacturing			0.00	(−0.01, 0.01)
Population			0.01	(0.00, 0.03)
Urban−rural division			2.11 ***	(0.50, 3.72)

** for *p* < 0.05 and *** for *p* < 0.01. If PM_1_ changes by 10 μg/m^3^, then the change in the incidence rate of female lung cancer relative to its mean = (10× PM_1_ coefficient)/mean incidence rate.

**Table 4:** Sensitivity analyses of ozone and PM_1_ effects to the control of the additional air pollutant (i.e., SO_2_).

Variables.	β	95% CI	Variables	β	95% CI
Ozone	3.95% ***	(2.89%, 14.84%)	PM_1_	3.47% **	(0.66%,14.92%)
Latitude	−1.60 ***	(−2.53, −0.66)	Latitude	−1.51 ***	(−2.45, −0.58)
Latitude^2^	0.03 ***	(0.02, 0.05)	Latitude^2^	0.03 ***	(0.02, 0.04)
Year 2015	−1.36 **	(−2.68, −0.05)	Year 2015	−0.69	(−2.21, 0.83)
Year 2016	−0.80	(−2.09, 0.49)	Year 2016	0.52	(−1.02, 2.07)
Finance	0.03 ***	(0.01, 0.06)	Finance	0.03 **	(0.01, 0.06)
Education	−1.02 ***	(−1.62, −0.43)	Education	−1.24 ***	(−1.84, −0.65)
Construction	−0.04 ***	(−0.06, −0.01)	Construction	−0.03 **	(−0.06, 0.00)
Manufacturing	−0.01	(−0.02, 0.00)	Manufacturing	0.00	(−0.01, 0.01)
Population	0.00	(−0.02, 0.02)	Population	0.01	(−0.01, 0.02)
Urban–rural division	2.37 ***	(0.74, 3.99)	Urban–rural division	2.41 **	(0.78, 4.04)
SO_2_	3.34% ***	(2.35%, 12.63%)	SO_2_	2.85% **	(0.87%, 11.89%)

** for *p* < 0.05 and *** for *p* < 0.01. When ozone, PM_1_ or SO_2_ changed by 10 μg/m^3^, the change in the incidence rate of female lung cancer relative to its mean = (10× coefficient for ozone, PM_1_ or SO_2_)/mean incidence rate.

**Table 5:** Sensitivity analysis of estimating effects of ozone, PM_1_ and the additional air pollutant (i.e., SO_2_) in the same model.

Variables.	Model 1		Model 2	
	β	95% CI	β	95% CI
Ozone	4.09% ***	(1.52%, 6.65%)	3.94% ***	(1.28%, 6.60%)
PM_1_	3.25% **	(0.16%, 6.34%)	3.45% **	(0.28%, 6.61%)
SO_2_	1.91% *	(−0.29%, 4.11%)	2.32% **	(−0.15%, 4.79%)
Latitude	−1.60 ***	(−2.50, −0.70)	−1.75 ***	(−2.69, −0.80)
Latitude^2^	0.03 ***	(0.02, 0.04)	0.03 ***	(0.02, 0.05)
Year 2015	−0.45	(−1.97, 1.07)	−0.53	(−2.05, 0.99)
Year 2016	0.30	(−1.25, 1.84)	0.16	(−1.40, 1.72)
Finance			0.03 ***	(0.01, 0.06)
Education			−1.13 ***	(−1.73, −0.53)
Construction			−0.03 ***	(−0.06, −0.01)
Manufacturing			−0.01	(−0.02, 0.00)
Population			0.00	(−0.01, 0.02)
Urban–rural division			2.39 ***	(0.77, 4.01)

* for *p* < 0.1, ** for *p* < 0.05 and *** for *p* < 0.01. When ozone, PM_1_ or SO_2_ changed by 10 μg/m^3^, the change in the incidence rate of female lung cancer relative to its mean = (10× coefficient for ozone, PM_1_ or SO_2_)/mean incidence rate.

**Table 6:** Effects of ozone and PM_1_ between urban and rural groups.

Groups	Ozone		PM_1_	
	β	95% CI	β	95% CI
Urban	2.77%	(−5.05%, 17.43%)	5.08% **	(−1.34%, 24.11%)
Rural	4.69% ***	(3.53%,17.49%)	3.52% **	(0.23%, 15.54%)

** for *p* < 0.05 and *** for *p* < 0.01. When ozone or PM_1_ changed by 10 μg/m^3^, the change in the incidence rate of female lung cancer relative to its mean = (10× coefficient for ozone or PM_1_)/mean incidence rate.

**Table 7:** Urban–rural modification effects on the association of health outcome with ozone and PM_1_.

Variables	β	95% CI	Variables	β	95% CI
Ozone	4.28% ***	(3.65%, 15.52%)	PM_1_	4.14% ***	(2.68%, 15.88%)
Latitude	−0.84 **	(−1.63, −0.05)	Latitude	−1.00 **	(−1.83, −0.18)
Latitude^2^	0.02 ***	(0.01, 0.03)	Latitude^2^	0.02 ***	(0.01, 0.03)
Year 2015	−1.32 **	(−2.64, 0.01)	Year 2015	−0.33	(−1.82, 1.16)
Year 2016	−0.87	(−2.16, 0.43)	Year 2016	0.90	(−0.62, 2.41)
Finance	0.03 ***	(0.01, 0.06)	Finance	0.03 **	(0.01, 0.06)
Education	−0.81 ***	(−1.38, −0.24)	Education	−1.24 ***	(−1.83, −0.64)
Construction	−0.04 ***	(−0.07, −0.01)	Construction	−0.03 **	(−0.06, 0.00)
Manufacturing	0.00	(−0.01, 0.01)	Manufacturing	0.00	(−0.01, 0.01)
Population	0.01	(−0.01, 0.03)	Population	0.01	(0.00, 0.03)
Ozone × Urban	1.01% **	(0.17%, 1.85%)	PM_1_×Urban	2.98% ***	(1.01%, 4.96%)

** for *p* < 0.05 and *** for *p* < 0.01. When ozone or PM_1_ changed by 10 μg/m^3^, the change in the incidence rate of female lung cancer relative to its mean = (10× coefficient for ozone or PM_1_)/mean incidence rate.

## Data Availability

Publicly available datasets were analyzed in this study. This data can be found here: https://weijing-rs.github.io/product.html (accessed on 2 August 2021).
